# Identification of Long Noncoding RNA Biomarkers for Hepatocellular Carcinoma Using Single-Sample Networks

**DOI:** 10.1155/2020/8579651

**Published:** 2020-11-14

**Authors:** Xiaoqing Yu, Jingsong Zhang, Rui Yang, Chun Li

**Affiliations:** ^1^School of Sciences, Shanghai Institute of Technology, Shanghai 201418, China; ^2^Key Laboratory of Systems Biology, State Key Laboratory of Cell Biology, Shanghai Institute of Biochemistry and Cell Biology, Center for Excellence in Molecular Cell Science, Chinese Academy of Sciences, Shanghai 200031, China; ^3^School of Mathematics and Statistics, Hainan Normal University, Haikou 571158, China

## Abstract

**Objective:**

Many studies have found that long noncoding RNAs (lncRNAs) are differentially expressed in hepatocellular carcinoma (HCC) and closely associated with the occurrence and prognosis of HCC. Since patients with HCC are usually diagnosed in late stages, more effective biomarkers for early diagnosis and prognostic prediction are in urgent need.

**Methods:**

The RNA-seq data of liver hepatocellular carcinoma (LIHC) were downloaded from The Cancer Genome Atlas (TCGA). Differentially expressed lncRNAs and mRNAs were obtained using the edgeR package. The single-sample networks of the 371 tumor samples were constructed to identify the candidate lncRNA biomarkers. Univariate Cox regression analysis was performed to further select the potential lncRNA biomarkers. By multivariate Cox regression analysis, a 3-lncRNA-based risk score model was established on the training set. Then, the survival prediction ability of the 3-lncRNA-based risk score model was evaluated on the testing set and the entire set. Function enrichment analyses were performed using Metascape.

**Results:**

Three lncRNAs (RP11-150O12.3, RP11-187E13.1, and RP13-143G15.4) were identified as the potential lncRNA biomarkers for LIHC. The 3-lncRNA-based risk model had a good survival prediction ability for the patients with LIHC. Multivariate Cox regression analysis proved that the 3-lncRNA-based risk score was an independent predictor for the survival prediction of patients with LIHC. Function enrichment analysis indicated that the three lncRNAs may be associated with LIHC via their involvement in many known cancer-associated biological functions.

**Conclusion:**

This study could provide novel insights to identify lncRNA biomarkers for LIHC at a molecular network level.

## 1. Introduction

Hepatocellular carcinoma, one of the most common cancers worldwide, is the third leading cause of worldwide mortality for various cancers, and its incidence rate per year remains increasing rapidly [1–3]. The risk factors for HCC include infection with hepatitis B virus (HBV) or hepatitis C virus (HCV), aflatoxin B1 intake, alcohol consumption, nonalcoholic fatty liver disease, and some hereditary diseases [4–6]. Since patients with HCC are usually diagnosed at late stages, when medication is no longer effective, understanding the molecular mechanisms of HCC and identifying biomarkers for early diagnosis and treatment seem to be essential [7, 8].

Long noncoding RNAs (lncRNAs) are non-protein-coding transcripts longer than 200 nucleotides. According to the well-known central dogma of molecular biology, genetic information is stored in protein-coding genes [9, 10], for which noncoding RNA (ncRNAs) have been considered as “junk genes” or “transcriptional noise” for a long time [11]. However, with the development of both experimental technology and computational methods, an increasing number of lncRNAs have been discovered in human transcriptome. Over the past decades, several researches have shown that lncRNAs are involved in almost the whole life cycle of cells through different mechanisms, and they have played diverse and important roles in many fundamental and critical biological processes, including transcriptional regulation, epigenetic regulation, organ or tissue development, cell differentiation and apoptosis, cell cycle control, metabolic processes, and chromosome dynamics [12–15]. Recently, several lncRNAs have been demonstrated to be associated with the development and survival in patients with different kinds of cancers, including HCC [16–18]. Moreover, many studies have highlighted the molecular mechanism and biological characters of lncRNAs in HCC occurrence and progression, and the result revealed that some lncRNAs can also serve as valuable prognostic predictors for HCC patients [19–21]. Despite precision medicine, which uses molecularly targeted therapy against malignant tumors and speeds up progress toward the discovery of novel molecular targets with the diagnostic and prognostic value [22], the management of patients with HCC remains problematic [23, 24]. Therefore, there is an urgent requirement to identify many more lncRNA biomarkers for HCC.

One key to achieving personalized medicine is to elucidate the molecular mechanisms of individual specific diseases, which generally result from the dysfunction of individual specific molecular network rather than the malfunction of single molecules. With rapid advances in high-throughput technologies, applying molecular networks to analyze human complex disease is attracting increasing wide attention [25, 26]. A molecular network, e.g., a gene regulatory network or a coexpression network, can be generally estimated by correlation coefficients of molecule pairs from expression or sequence data of multiple samples [27, 28]. In recent years, based on biological and clinical data, a number of network-based methods were proposed not only to identify disease modules and pathways but also to elucidate molecular mechanisms of disease development at the network level [29–31]. Many studies have shown that network-based biomarkers are superior to traditional single-molecule biomarkers for accurately characterizing disease states due to their additional information on interactions and networks [28, 30, 32, 33]. In particular, a single-sample network is considered to be reliable for accurately characterizing the specific disease state of an individual. It can be directly used to identify the biomarkers and further elucidate the molecular mechanisms of a disease for individual patients [29].

In this study, we aimed to identify the lncRNA biomarkers for the patients with LIHC based on the RNA-seq data from TCGA. By constructing the single-sample networks for the 371 tumor patients, we obtained three lncRNA biomarkers associated with overall survival (OS) of LIHC patients. Then, a 3-lncRNA-based risk score model was established on the training set, which could effectively predict the OS of LIHC patients. The independence and the predictive ability for survival prediction of the 3-lncRNA-based risk score were validated on the testing set and the entire set.

## 2. Materials and Methods

### 2.1. Dataset

By using GDC API (https://gdc.cancer.gov/developers/gdc-application-programming-interface-api), RNA sequencing data and clinical information from individuals with liver hepatocellular carcinoma (LIHC) were achieved from TCGA (https://portal.gdc. http://cancer.gov/projects/TCGA-LIHC), which included 371 tumor samples and 50 normal samples.

### 2.2. Differential Expression Analysis

EdgeR is a Bioconductor package for differential expression analysis of replicate count data, which was widely used in the differential expression analysis of high-throughput sequencing data. In our study, we extracted the expression profile of mRNAs and lncRNAs from RNA-seq count data, which was normalized using the edgeR package (version 3.22.5). Those mRNAs and lncRNAs with zero expression value in more than 10% samples were discarded. The differentially expressed lncRNAs and mRNAs were calculated by edgeR at a threshold of FDR < 0.05 and ∣log_2_(foldchange) | >1.

### 2.3. Construction of the Single-Sample Networks

In our study, the differentially expressed mRNAs and lncRNAs which had the same gene names were removed. As a result, 3329 differentially expressed mRNAs and 956 differentially expressed lncRNAs for each sample were left for further investigation. A single-sample network was constructed based on statistical perturbation analysis of a single test sample against a group of given reference samples, which can accurately characterize the disease state of an individual or a sample. The more detailed description about the single-sample network can be found in Ref. [34]. In our study, we took 50 normal samples as the reference samples, while 371 tumor samples were the test samples. Firstly, based on the gene expression data of the reference samples, a reference correlation network can be constructed by computing the Pearson correlation coefficient (PCC) between lncRNA-lncRNA and lncRNA-mRNA pairs, which was denoted as *N*_*r*_. Then, adding a test sample *s* to the reference samples, another perturbed correlation network was obtained in the same way, which was denoted as *N*_*p*_. By comparing the difference of the two correlation networks, we can get a single-sample network *N*_ssn_ for this test sample *s*(*N*_ssn_ = |*N*_*r*_ − *N*_*p*_|). Finally, 371 single-sample networks were obtained in our study.

For convenience, we transformed each single-sample network to an adjacency matrix Δ*D*, and the element Δ*D*_*i*,*j*_ represents the ΔPCC of the edge for a pair of molecules in the single-sample network. As the theoretical foundation for this method, if there were obvious differences between the reference samples and the single sample *s* in terms of the gene expression pattern, adding the tumor sample *s* to the reference samples would cause significant changes of the PCC on some edges in the perturbed network. We assumed that if a lncRNA might be a key biomarker, the sum of the ΔPCC of the edges linked by the lncRNA would be higher than others. Then, a vector SD was used to represent the sum of ΔPCC of the edges linked by each lncRNA in a single-sample network, which was denoted as

(1) $$\begin{array}{c} \text{SD}={\left({\text{SD}}_{1},{\text{SD}}_{2},\cdots ,{\text{SD}}_{i},\cdots ,{\text{SD}}_{956}\right)}^{T}, \end{array}$$

where SD_*i*_ is the sum of ΔPCC of all the edges linked by the *i*th lncRNA in a single-sample network and can be calculated by

(2) $$\begin{array}{c} {\text{SD}}_{i}=\underset{j}{\sum }\Delta D_{ij}\left(i=1,2,\cdots ,956,j=1,2,\cdots ,4285\right). \end{array}$$

Consequently, all the 371 single-sample networks can be represented by a matrix *M*_956×371_:

(3) $$\begin{array}{c} M=\left[\begin{array}{cccccc} {\text{SD}}_{1,1} & {\text{SD}}_{1,2} & \cdots & {\text{SD}}_{1,j} & \cdots & {\text{SD}}_{1,371}\\ {\text{SD}}_{2,1} & {\text{SD}}_{2,2} & \cdots & {\text{SD}}_{2,j} & \cdots & {\text{SD}}_{2,371}\\ \cdots & \cdots & \cdots & \cdots & \cdots & \cdots \\ {\text{SD}}_{i,1} & {\text{SD}}_{i,2} & \cdots & {\text{SD}}_{i,j} & \cdots & {\text{SD}}_{i,371}\\ \cdots & \cdots & \cdots & \cdots & \cdots & \cdots \\ {\text{SD}}_{956,1} & {\text{SD}}_{956,2} & \cdots & {\text{SD}}_{956,j} & \cdots & {\text{SD}}_{956,371} \end{array}\right],\left(\begin{array}{c} i=1,2,\cdots ,956\\ j=1,2,\cdots ,371 \end{array}\right), \end{array}$$

where *i* denotes the *i*th lncRNA and *j* denotes the *j*th tumor sample.

**Figure 1:**
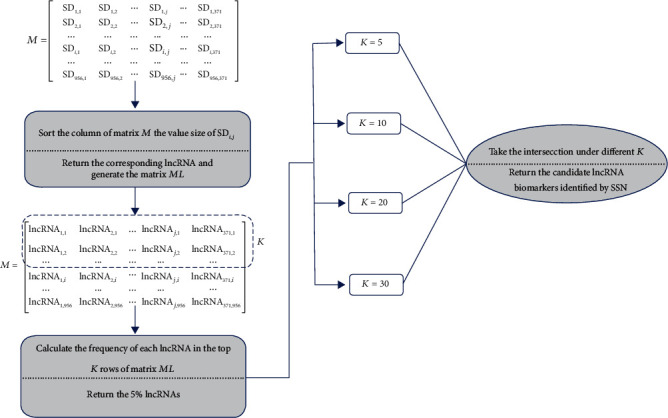
The ranking system for identifying the candidate lncRNA biomarkers by SSN.

Different from the method in Ref. [34], we designed a ranking system to identify the candidate lncRNA biomarker according to the matrix *M*. Firstly, each column in matrix *M* was sorted by the value size of SD_*ij*_, and the items in the column returned to the corresponding lncRNA in matrix *ML*. Then, we calculated the frequency of each lncRNA in the top *K* rows of matrix *ML* and the top 5% lncRNAs were retained. Finally, in order to improve the effectiveness of our method, we took the intersection of the candidate lncRNAs under different *K* (*K* = 5,10,20,30) as the final candidate lncRNA biomarkers by SSN. The flowchart of the ranking system is shown in Figure 1.

### 2.4. Survival Analysis

The differentially expressed lncRNAs, of which the expression level was zero that exceeded 10% of all subjects, were removed from the prognostic analysis. In the meantime, clinicopathological features, including survival information, were also achieved from TCGA. Samples without sufficient clinical data were omitted. Finally, the prognostic analysis included a total of 307 tumor samples with the expression data from 956 lncRNAs. The univariate and multivariate Cox regression analyses were conducted to evaluate the association between the variables and the OS of LIHC patients on the training set, the testing set, and the entire set, and statistical significance was assessed using *p* < 0.05. The Kaplan-Meier plots were employed to observe the prognosis effect. The ROC curve analysis was used to evaluate the prognosis performance for 5-year survival rate. All analyses were performed on the R3.4.3 framework.

### 2.5. Construction of the Risk Score Model

The 307 patients were randomly divided into two datasets. 154 patients were used as the training set to build a risk score model based on the candidate lncRNA biomarkers, while the other 153 patients were used as the testing set to evaluate the predictive ability of the risk score model. Firstly, the multivariate Cox regression analysis was used to evaluate the association between the expression of the lncRNA biomarkers and the patient's OS in the training set. Then, a risk score model was built by linear combination of the expression levels of the lncRNA biomarkers weighted by their Cox regression coefficients. The calculation formula of the risk score model was as follows:

(4) $$\begin{array}{c} \text{Risk Score}\left(\text{RS}\right)=\overset{n}{\underset{i=1}{\sum }}\left({\text{Exp}}_{i}\times {\text{Coe}}_{i}\right), \end{array}$$

where *i* represents the total number of the lncRNA biomarkers, Exp indicates the expression profiles of lncRNA, and Coe is the estimated regression coefficient of the *i*th lncRNA derived from the multivariate Cox analysis. Based on this formula, each patient with LIHC had an RS and the median RS was treated as a cut-off point to divide the patients into the low-risk group and the high-risk group.

### 2.6. Pathway and Functional Enrichment Analysis

Metascape (http://metascape.org/gp/index.html) is a free gene annotation and analysis resource that helps biologists make sense of one or multiple gene lists [35]. To understand the function of the identified lncRNA biomarkers in our study, the Gene Ontology (Go) and Kyoto Encyclopedia of Genes and Genomes (KEGG) pathway enrichment analysis was performed by Metascape. A value of *p* < 0.01 was set as the cut-off for significance.

## 3. Results

### 3.1. Differentially Expressed lncRNAs and mRNAs in LIHC

**Figure 2:**
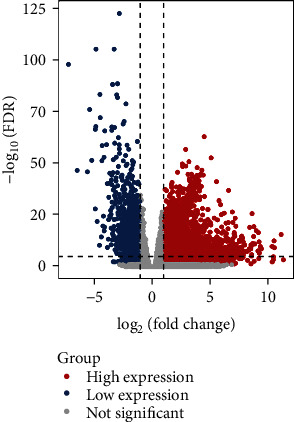
The volcano plot of the differentially expressed mRNAs and lncRNAs. Red indicates high expression, blue indicates low expression, and grey indicates nondifferential expression. The *x*-axis represents a log_2_FC and the *y*-axis represents the -log_10_(FDR).

We downloaded the expression data of 60483 RNA, from which 14822 lncRNAs and 19814 mRNAs were obtained by the gene type data reported by the genome GRCh38.p13. By calculating with edgeR, a total of 956 lncRNAs and 3329 mRNAs were considered to be differentially expressed between tumor samples and normal samples. The volcano plot of the differentially expressed mRNAs and lncRNAs is shown in Figure 2.

### 3.2. The Candidate lncRNAs Identified by Single-Sample Networks

In our study, the top 48 lncRNAs (5% of 956 lncRNAs) that had the highest frequency of occurrence in all the 371 tumor samples remained as the candidate lncRNA biomarkers by the ranking system. Furthermore, by taking the intersection of the candidate lncRNAs identified under different *K*(*K* = 5, 10, 20, 30), 27 lncRNAs were obtained as the final candidate lncRNA biomarkers by the ranking system according to 371 single-sample networks. The detailed differential expression information of these lncRNAs is listed in Table S1.

### 3.3. Identification of Prognostic lncRNA Biomarkers

**Figure 3:**
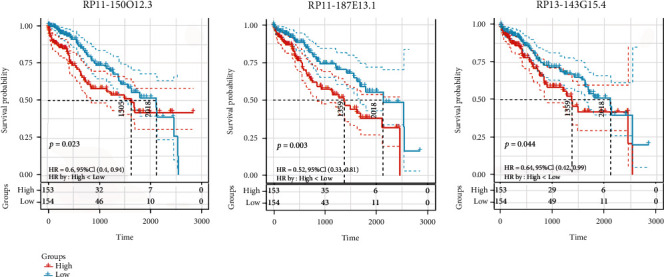
Kaplan-Meier curves of the survival probability of the three lncRNAs.

Hypothesized that the candidate lncRNA biomarkers selected by SSN might be involved in LIHC development and progression, we examined whether these lncRNAs were associated with the survival of patients with LIHC. The univariate survival analysis was performed based on the expression value of these lncRNAs and the OS of the LIHC patients. The follow-up period of these patients involved was 5 years (1825 days). According to the expression of the three lncRNA biomarkers, 307 patients were divided into high expression group (*n* = 154) and low expression group (*n* = 153). The results showed that of all the 27 lncRNAs identified by SSN, three lncRNAs (RP11-150O12.3, RP11-187E13.1, and RP13-143G15.4) had significant prognostic differences (*p* < 0.05). The Kaplan-Meier plots employed to observe the prognosis effect of the three lncRNAs are presented in Figure 3. The detailed description of the three lncRNAs is shown in Table 1.

### 3.4. Construction of Risk Score Model in the Training Set

**Figure 4:**
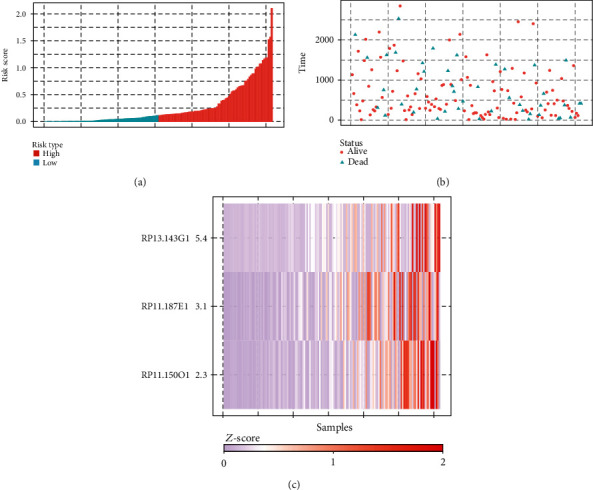
Construction of the 3-lncRNA-based risk score model. (a, b) The risk score distribution and survival status of the patients in the training set. (c) Heat map of the expression levels of the three lncRNAs.

**Figure 5:**
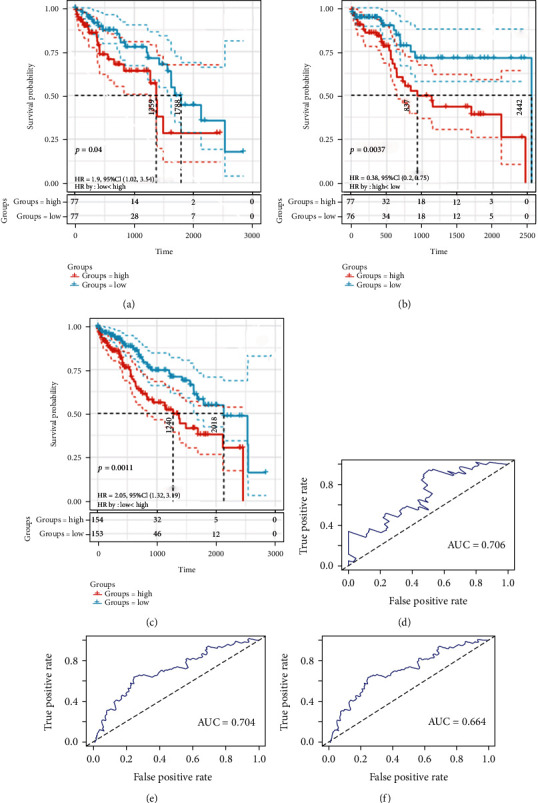
Kaplan-Meier survival analysis and ROC curve analysis of the 3-lncRNA-based risk score model. (a–c) Kaplan-Meier survival analysis in the training set, the testing set, and the entire set. (d–f) 5-year ROC curve analysis for survival prediction in the training set, the testing set, and the entire set.

By using the multivariate Cox regression analysis in the training set, based on the expression value of the three lncRNAs and the regression coefficients, a 3-lncRNA-based risk score model was constructed as follows: riskscore(RS) = 0.5324∗expressionlevelofRP11 − 150O12.3 + 0.7105∗expressionlevelofRP13 − 143G15.4 + 0.3738∗expressionlevelofRP11 − 187E13.1. Based on the above risk score formula, the RS of each patient in the training set was calculated. The 154 patients in the training set were divided into two groups of low-risk group (*n* = 77) and high-risk group (*n* = 77) according to the median point of the risk score. The risk distribution and vital status of 154 patients and the expression heat map of the 3 lncRNAs are presented in Figure 4. The Kaplan-Meier survival analysis displayed that the survival of LIHC patients in the high-risk group was significantly shorter than that in the low-risk group (Figure 5(a), *p* = 0.04). The median survival of patients in the high-risk group and the low-risk group was 3.72 years and 4.89 years, respectively. To assess the prognostic performance of the risk score, a 5-year ROC curve analysis was performed. The result showed that the 3-lncRNA-based risk score model had a good prediction efficiency in the training set (Figure 5(d), AUC = 0.706).

### 3.5. Evaluation of the Risk Score Model in the Testing Set and the Entire Set

To assess the robustness of the 3-lncRNA-based risk score model in OS prediction for LIHC patients, we further examined it in the testing set and the entire set. The same 3-lncRNA-based risk score model and cut-off point derived from the training set were used to divide the testing set and the entire set into the high-risk group and the low-risk group (*n* = 77 and 77, *n* = 154 and 153, respectively). In the testing set, the Kaplan-Meier curve showed that the survival of LIHC patients in the low-risk group exhibited a longer OS as compared to those in the high-risk group (Figure 5(b), *p* = 0.0037). The median survival of patients in the high-risk group and the low-risk group was 2.29 years and 6.69 years, respectively. In the entire set, a similar result was shown that patients in the high-risk group exhibited a shorter OS as compared to those in the low-risk group (Figure 5(c), *p* = 0.0011). The median survival of patients in the high-risk group and the low-risk group was 3.39 years and 5.52 years, respectively. The AUC of 5-year ROC curve in the testing set and the entire set was 0.704 and 0.664, implying a good prognostic capacity of the 3-lncRNA-based risk score (Figures 5(e) and 5(f)).

### 3.6. Independence of the 3-lncRNA-Based Risk Score for Survival Prediction

To evaluate the independent prediction performance of the three lncRNAs when considering the traditional clinical factors, univariate and multivariate Cox regression analyses were conducted on the training set, the testing set, and the entire set. For each dataset, the explanatory variables included 3-lncRNA-based risk score, age, sex, weight, grade, and tumor stage. In the multivariate Cox regression analysis, the above factors were regarded as covariates, while the OS was used as a dependent variable. The results showed that the 3-lncRNA-based risk score was significantly associated with the OS of patients (HR = 0.416, 95% CI [0.207-0.836], *p* = 0.014 for the training set; HR = 0.459, 95% CI [0.225-0.936], *p* = 0.032 for the testing set; HR = 0.498, 95% CI [0.313-0.792], *p* = 0.003 for the entire set), which revealed that the 3-lncRNA-based risk score was an independent predictor of OS for LIHC patients. More detailed results are provided in Table 2.

### 3.7. Pathway and Functional Enrichment Analysis

**Figure 6:**
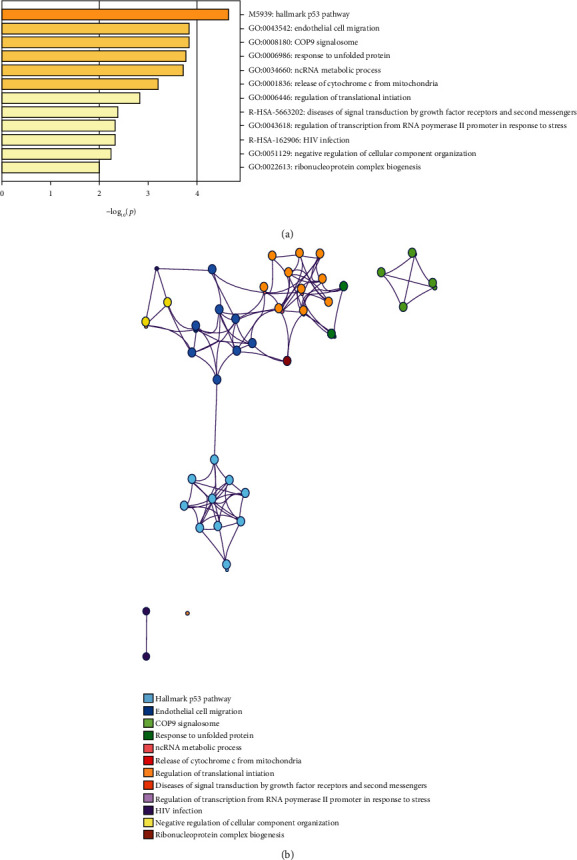
Pathway and process enrichment analysis. (a) KEGG functional analysis of the coexpressed genes of the three lncRNAs. (b) Detailed net structure of the coexpressed genes of the three lncRNAs.

We were also interested in the molecular mechanisms of the three lncRNAs. Unfortunately, little publication was found on the functional mechanism of these lncRNAs. Finally, we performed Pearson correlation analyses between the three lncRNAs and protein-coding genes based on their expression levels in TCGA LIHC cohort. The protein-coding genes that correlated with at least 1 of the three lncRNAs (Pearsoncoefficient > 0.45, *p* < 0.01) were considered to be significantly correlated coexpressed genes of the three lncRNAs, which are listed in Table 3. Then, pathway and process enrichment analyses were carried out with the following ontology sources in Metascape: KEGG pathways and GO biological processes. All genes in the genome were used as the enrichment background. Terms with *p* < 0.01, minimumcount = 3, and enrichmentfactor > 1.5 were collected and grouped into clusters based on membership similarities. We found that the coexpressed genes of three lncRNAs were mainly associated with terms related to endothelial cell migration, ncRNA metabolic process, cell cycle arrest, etc. (Figure 6). The retrieved data is also provided in Table S2.

## 4. Discussion

HCC is the most common type of liver cancer, accounting for about 80% of the tumours in this organ. Although there are advances in the diagnosis and treatment of HCC, the OS time of patients with HCC remains poor. Recently, molecular biomarkers have been identified throughout the various clinical stages and pathological tissue types, and multiple studies have revealed that differential expression of lncRNAs played critical roles in cancer development, indicating their potential as novel biomarkers for cancer diagnosis and prognosis [36, 37]. However, until now, only a few lncRNAs had been experimentally verified for HCC. Thus, it is necessary to identify more potential lncRNA biomarkers for HCC.

In the present study, we analyzed the expression profiles and clinical information of LIHC patients from TCGA database. The differential expressed lncRNAs and mRNAs were screened to construct the single-sample networks for 371 LIHC patients. According to the 371 single-sample networks, 27 candidate lncRNAs were selected by the ranking system designed for identifying the lncRNA biomarkers from the single-sample networks. Furthermore, by using the univariate Cox regression, three lncRNAs (RP11-150O12.3, RP11-187E13.1, and RP13-143G15.4) were selected as the lncRNA biomarkers associated with patients' OS. Next, multivariate Cox regression was performed in the training set to establish a 3-lncRNA-based risk score model based on the expressions and relative contributions in the multivariate Cox regression model. The 3-lncRNA-based risk score had a good prognosis prediction ability for OS of patients with LIHC, which was tested in the testing set and the entire set. Moreover, we performed multivariate Cox regression analyses using the 3-lncRNA-based risk score and other clinical data in the training set, the testing set, and the entire set. The result showed that the 3-lncRNA-based risk score was an independent predictor for the OS of LIHC patients.

The results of function enrichment analysis showed that these KEGG pathways and functional categories of the three lncRNA biomarkers were all closely associated with tumorigenesis. For instance, the transcription factor p53 is a tumor suppressor, activating downstream targets to trigger cell cycle arrest and apoptosis [38], and several lncRNAs participate in the p53 regulatory network and serve as p53 regulators or effectors [39]. Moreover, a recent global transcriptome study identified that sixteen p53 target lncRNAs forming a pathway web constitute tumor suppressor signature with high diagnostic power [40]. Importantly, Jelena et al. revealed that p53 might play multifunctional roles in different stages in HCC [41]. Moreover, the metastatic characteristics of liver cancer are the key factors affecting the survival and prognosis of tumor patients, and the process of cell migration is involved in tumor metastasis [42]. The COP9 signalosome is a highly conserved protein complex implicated in diverse biological functions that involve ubiquitin-mediated proteolysis [43]. Also, research demonstrates that COP9 signalosome is an important regulator of cell cycle and cell survival mediating the proliferation of HCC cells and highlight that COP9 signalosome might be a promising strategy for anti-HCC therapy [44]. The unfolded protein response is a signal transduction cascade, which acts as a quality control mechanism for protein synthesis and consequently can act to protect cells from an adverse external microenvironment. The study declared that the unfolded protein response is activated in the majority of HCC and may play a role in chemoresistance to the most widely used chemotherapy agent, doxorubicin, and have effects on newer antiestrogen and multikinase inhibitor therapies [45]. The present results indicated that the three lncRNAs might have an important role in LIHC via their involvement in these known cancer-associated biological functions.

Besides, previous studies have demonstrated that RP11-150O12.3 was significantly and independently associated with survival of HCC patients [46]. Moreover, RP11-150O12.3 also has been recognized as an independent predictor of gastric cancer prognosis [47], which also related to survival time of patients with colorectal adenocarcinoma [48]. RP13-143G15.3 is abnormally expressed in liver cancer and may act as an important role of tumor suppressor in human HCC [49]. The results of these studies provided some grounding to validate our findings.

In our study, the single-sample networks were constructed for identifying the lncRNA biomarkers associated with LIHC patients. Actually, a single-sample network in this study is not a real molecular network for each sample but a perturbation network for a single sample against the reference network. It mainly reflects the variation between normal and disease samples in terms of interactions or a network. Similarly, a differential expression of a gene is not the real gene expression level for each sample but the variation of the gene expression between normal and disease samples. The advantage of the single-sample network is that it cannot only characterize the personalized features for all the single samples but also can be directly applied to the data analysis of single samples at the molecular network level, which is superior to traditional single molecular biomarker.

There are several limitations to the present study. First, the primary purpose of the present study was to identify lncRNA biomarker for LIHC at the molecular network level. The lncRNA biomarkers selected in our study should be further investigated incorporating more specific clinical characteristics to fully understand the associations involved. Second, the three lncRNA biomarkers were only validated in the datasets from TCGA database, which required additional confirmation in other large cohorts of LIHC patients in the future. Third, the lncRNA biomarkers identified in our study have no validation in fresh samples and experiment studies.

## 5. Conclusion

In this study, we constructed the single-sample networks for LIHC patients and identified three lncRNA biomarkers associated with the OS of LIHC patients. A 3-lncRNA-based risk score model with the ability to effectively predict the survival of LIHC patients was established, which was validated to be an independent predictor for the OS of LIHC patients. Functional enrichment analysis revealed that three lncRNAs may be associated with LIHC via their involvement in many known cancer-associated biological functions. This study provides a new perspective for identifying the lncRNA biomarkers at the molecular network level.

## Figures and Tables

**Table 1:** The three lncRNAs significantly associated with OS of LIHC patients.

No.	Gene symbol	Ensemble ID	Chromosome	Hazard ratio	*p* value
1	RP11-150O12.3	ENSG00000254290.1	Chr.8:37,597,480-37,599,858	0.61 (0.4-0.94)	0.023
2	RP11-187E13.1	ENSG00000258474.1	Chr.14:31,944,853-31,950,382	0.52 (0.33-0.81)	0.003
3	RP13-14G15.4	ENSG00000237596.2	Chr.6:136415853-136546733	0.64 (0.42-0.99)	0.044

**Table 2:** Univariate and multivariate Cox proportional hazards regression analysis.

Variables	Univariate analysis			Multivariate analysis		
	HR	95% CI	*p* value	HR	95% CI	*p* value
Training set (*n* = 154)						
^a^Risk score (high vs. low)	0.526	0.282-0.980	0.043	0.416	0.207-0.836	0.014
Age	1.031	1.006-1.058	0.015	1.029	0.998-1.059	0.061
Sex (male vs. female)	0.851	0.462-1.566	0.605	1.077	0.519-2.236	0.841
Weight	0.999	0.983-1.016	0.939	0.997	0.981-1.014	0.772
Grade (I+II vs. III+IV)	0.138	0.312-0.607	0.009	0.352	0.032-1.524	0.290
Stage (I+III vs. II+IV)	0.379	0.143-1.000	0.050	0.730	0.189-2.830	0.670
Testing set (*n* = 153)						
^a^Risk score (high vs. low)	0.384	0.197-0.751	0.003	0.459	0.225-0.936	0.032
Age	1.025	0.998-1.053	0.069	1.023	0.994-1.052	0.114
Sex (male vs. female)	0.605	0.325-1.126	0.113	0.711	0.361-1.397	0.322
Weight	0.999	0.983-1.017	0.971	0.999	0.983-1.016	0.979
Grade (I+II vs. III+IV)	1.242	0.166-9.313	0.833	0.510	0.180-1.441	0.996
Stage (I+III vs. II+IV)	0.478	0.204-1.123	0.090	0.675	0.220-2.068	0.204
Entire set (*n* = 307)						
^a^Risk score (high vs. low)	0.487	0.313-0.756	0.001	0.498	0.313-0.792	0.003
Age	1.028	1.010-1.047	0.002	1.023	1.004-1.043	0.017
Sex (male vs. female)	0.715	0.463-1.105	0.131	0.811	0.506-1.299	0.384
Weight	0.999	0.988-1.011	0.930	0.999	0.988-1.011	0.966
Grade (I+II vs. III+IV)	0.436	0.134-1.418	0.168	0.582	0.116-2.910	0.510
Stage (I+III vs. II+IV)	0.427	0.226-0.804	0.008	0.570	0.256-1.269	0.169

Notes. ^a^Derived from the 3-lncRNA-based risk score model.

**Table 3:** The coexpressed genes of three lncRNAs.

Key lncRNA	Coexpressed gene
RP11-150O12.3	CLK1, BAG3, MXD1, GRIK1, TNFSF9, DNAJA4, FAM83A, FOXL1, PPP1R15A, SNORD14E, HSPA6, FOXC2
RP13-143G15.4	ZNF703, TMCC1, NKD1, RNF43, CYB5B
RP11-187E13.1	UFSP1, HN1, C7orf61, LSM8, AP1S1, ELOB, TSR3, PSMG3, COPS9, COPS6, ZNF593, LOC101927420, CLDND2, ST7-AS1, C6orf52, LOC106660606, B9D1, FIS1, PRSS3, S100A13, S100A16, NSUN5, LMNA, LAGE3, ACD, SNHG19, HSPB1, TMEM54, LINC00896, TMSB10, SFN, BRI3, EIF3B, HRAS, MRPL28, MIR6132, NT5C, PTCD1, RASSF1-AS1, MINCR, LOC105371849, METTL26, DPM3, BCL7C, NUDT1, AP1M2, MCM7, BOLA2B, JOSD2, LOC400684, PUSL1, ARF4-AS1, UQCC3, POLR2J, NAA10, LOC101928659, PGP, S100P, POP7, LAMTOR4

## Data Availability

The data used to support the findings of this study are available from the corresponding author upon request.

## Abbreviations

LIHC: Liver hepatocellular carcinoma
HCC: Hepatocellular carcinoma
HBV: Hepatitis B virus
HCV: Hepatitis C virus
lncRNA: Long noncoding RNA
OS: Overall survival
PCCs: Pearson correlation coefficients
SSN: Single-sample network
Go: Gene Ontology
KEGG: Kyoto Encyclopedia of Genes and Genomes.

## Supplementary Materials

Supplementary 1 Table S1: list of 27 differentially expressed lncRNAs in LIHC.

Supplementary 2 Table S2: the results of GO and KEGG enriched terms.
