# Supplementary material for: Identification of Long Noncoding RNA Biomarkers for Hepatocellular Carcinoma Using Single-Sample Networks
Source: Biomed Res Int. 2020 Nov 14;2020:8579651. doi: 10.1155/2020/8579651 (PMC7700720; doi:10.1155/2020/8579651)
Supplement: Supplementary 1 — Table S1: list of 27 differentially expressed lncRNAs in LIHC. [file 8579651.f1.docx]

Table S1: List of 27 differentially expressed LncRNAs in LIHC

| Symbol | Log2(FC) | regulated |
| --- | --- | --- |
| RP13-143G15.4 | 4.43176756 | Up-regulated |
| RP11-150O12.3 | 3.04785636 | Up-regulated |
| RP11-65J21.4 | 2.95793062 | Up-regulated |
| AC007099.1 | 5.1090639 | Up-regulated |
| RP3-323A16.1 | 2.98752764 | Up-regulated |
| RP11-184M15.1 | 2.21223026 | Up-regulated |
| CTD-2263F21.1 | 3.23911878 | Up-regulated |
| RP11-923I11.6 | 1.87016107 | Up-regulated |
| AP000569.9 | 2.32955155 | Up-regulated |
| RP3-460G2.2 | 2.43448011 | Up-regulated |
| RP11-187E13.1 | 3.47230266 | Up-regulated |
| CTD-2616J11.16 | 2.74269103 | Up-regulated |
| RP11-165A20.3 | 1.72389288 | Up-regulated |
| RP11-304F15.3 | 1.42538627 | Up-regulated |
| RP11-16E12.2 | 2.49858036 | Up-regulated |
| KB-1572G7.3 | 1.57076376 | Up-regulated |
| RP11-115D19.1 | 3.55727645 | Up-regulated |
| RP11-260M19.2 | -1.6083415 | Down-Regulated |
| RP11-676J12.7 | -2.4390932 | Down-Regulated |
| RP11-474P2.4 | -1.2818421 | Down-Regulated |
| VIPR1-AS1 | -1.3076539 | Down-Regulated |
| RP11-772C9.1 | -1.8401121 | Down-Regulated |
| RP11-214O1.3 | -1.2716604 | Down-Regulated |
| RP11-830F9.5 | -2.2801353 | Down-Regulated |
| AP002954.4 | -1.7468972 | Down-Regulated |
| RP11-442O1.3 | -1.2720914 | Down-Regulated |
| CTD-2154B17.4 | -1.120461 | Down-Regulated |
