# Supplementary material for: Identification of Long Noncoding RNA Biomarkers for Hepatocellular Carcinoma Using Single-Sample Networks
Source: Biomed Res Int. 2020 Nov 14;2020:8579651. doi: 10.1155/2020/8579651 (PMC7700720; doi:10.1155/2020/8579651)
Supplement: Supplementary 2 — Table S2: the results of GO and KEGG enriched terms. [file 8579651.f2.docx]

Table S2: The results of GO and KEGG enriched terms

| Term | Description | count | Log10(P) |
| --- | --- | --- | --- |
| M5939 | Hallmark P53 Pathway | 6 | -4.66 |
| GO: 0043542 | Endothelial cell migration | 6 | -3.85 |
| GO:0008180 | COP9 signalosome | 3 | -3.84 |
| GO:0006986 | Response to unfolded protein | 5 | -3.77 |
| GO:0034660 | ncRNA metabolic process | 8 | -3.72 |
| GO:0001836 | Release of cytochrome c from mitochondria | 3 | -3.20 |
| GO:0006446 | Regulation of translational initiation | 3 | -2.83 |
| R-HAS-5663202 | Diseases of signal transduction by growth factor receptors and second messengers | 5 | -2.37 |
| GO:0043618 | Regulation of transcription from RNA polymerase II promoter in response to stress | 3 | -2.33 |
| R-HAS-162906 | HIV infection | 4 | -2.31 |
| GO:0051129 | Negative regulation of cellular component organization | 7 | -2.24 |
| GO:0022613 | Ribonucleprotein complex biogenesis | 5 | -2.00 |
